# A distinct lineage of *Caudovirales* that encodes a deeply branching multi-subunit RNA polymerase

**DOI:** 10.1038/s41467-020-18281-3

**Published:** 2020-09-09

**Authors:** Alaina R. Weinheimer, Frank O. Aylward

**Affiliations:** Department of Biological Sciences, Virginia Tech, Blacksburg, VA 24061 USA

**Keywords:** Phylogenetics, Viral genetics, Bacteriophages, Viral evolution

## Abstract

Bacteriophages play critical roles in the biosphere, but their vast genomic diversity has obscured their evolutionary origins, and phylogenetic analyses have traditionally been hindered by their lack of universal phylogenetic marker genes. In this study we mine metagenomic data and identify a clade of *Caudovirales* that encodes the β and β′ subunits of multi-subunit RNA polymerase (RNAP), a high-resolution phylogenetic marker which enables detailed evolutionary analyses. Our RNAP phylogeny revealed that the *Caudovirales* RNAP forms a clade distinct from cellular homologs, suggesting an ancient acquisition of this enzyme. Within these multimeric RNAP-encoding *Caudovirales* (mReC), we find that the similarity of major capsid proteins and terminase large subunits further suggests they form a distinct clade with common evolutionary origin. Our study characterizes a clade of RNAP-encoding *Caudovirales* and suggests the ancient origin of this enzyme in this group, underscoring the important role of viruses in the early evolution of life on Earth.

## Abstract (web summary)

Viruses have been difficult to position in the Tree of Life using phylogenetic methods. This study uses an ancient enzyme multi-subunit RNA polymerase (RNAP) to reveal a novel viral group, the *Caudovirales*, and to suggest an ancient origin of RNAP in this group.

## Introduction

The creation of a Tree of Life (TOL) that encompasses all life forms on Earth has been a central goal of Biology ever since the theory of evolution was introduced by Darwin and Wallace in the nineteenth century^1^. In recent decades, efforts toward the creation of a TOL has progressed considerably due to advances in molecular phylogenetic methods, the increased availability of whole-genome sequencing datasets, and the identification of highly conserved marker genes that are present in all cellular life and can be readily compared in molecular phylogenetic trees^2–4^. Although the TOL has been a useful concept for studying cellular lineages, it has proven more problematic for viruses, which lack the necessary phylogenetic marker genes that would allow their inclusion into typical molecular phylogenies of cellular life^5^. Indeed, current frameworks for dealing with viruses generally group them together as capsid-encoding organisms, to distinguish them from cellular ribosome-encoding organisms, a framework that explicitly classifies them due to their lack of the ribosomal genes that are commonly used for constructing molecular phylogenies^6^.

There are high-resolution phylogenetic marker genes other than ribosomal genes that can provide insight into deep evolutionary relationships, however, such as the multi-subunit DNA-directed RNA polymerase (RNAP). RNAP is an ancient enzyme present in all Bacteria, Archaea, and Eukarya, and it has often been used to provide high-resolution phylogenies of divergent microbial lineages^7–10^. Importantly, although viruses lack ribosomal genes, some viruses encode their own copy of RNAP that can be used to evaluate their evolutionary relationships to cellular life; e.g., one recent study focusing on Nucleo-Cytoplasmic Large DNA Viruses (NCLDV) analyzed RNAP phylogenies and found evidence that this viral group emerged prior to modern Eukaryotes^11^.

Multi-subunit RNAP is composed of two core subunits referred to as the β- and β′-subunits in Bacteria, RPB1 and RPB2 subunits in Eukarya, and B and A in Archaea, respectively. These two subunits are homologous and likely originate from an ancient duplication event^12^. Several bacteriophages have been found to encode RNAPs that have likely been acquired in distinct ways. For example, the recently discovered crAssphage, which are widespread in the human microbiome, encode an RNAP enzyme in which the β- and β′-subunits are fused into one protein^13^, but this enzyme is highly divergent from cellular RNAP subunits and sequence homology is not easily identified. Moreover, some bacteriophage have been shown to encode a single-subunit YonO protein, which shares homologous motifs with the β′-subunit of RNAP and functions as a DNA-dependent RNAP^14^, but these enzymes are also highly divergent compared to cellular homologs. In addition, a recent large-scale metagenomic analysis identified the presence of multi-subunit RNAP homologs in contigs from environmental bacteriophage, suggesting that the prevalence of this enzyme in viruses may be broader than previously thought^15^.

In this study, we surveyed multiple large DNA sequence datasets to identify the occurrence of RNAP in bacteriophage genomes and examine the evolutionary links between these enzymes and cellular homologs. As the vast majority of viral diversity remains uncultivated, we focused our analysis on viral sequences present in metagenomic datasets and ultimately identified 97 bacteriophage-encoded RNAP that we used for subsequent analysis. Phylogenetic analyses of the RNAP encoded by these bacteriophages suggests that they are distinct from cellular RNAP and are the result of an ancient acquisition. Moreover, analysis of other marker genes suggest these viruses belong to a lineage of *Caudovirales*, which we refer to as multi-subunit RNAP-encoding *Caudovirales* (mReC).

## Results

### Detection of RNAP-encoding *Caudovirales*

**Fig. 1:**
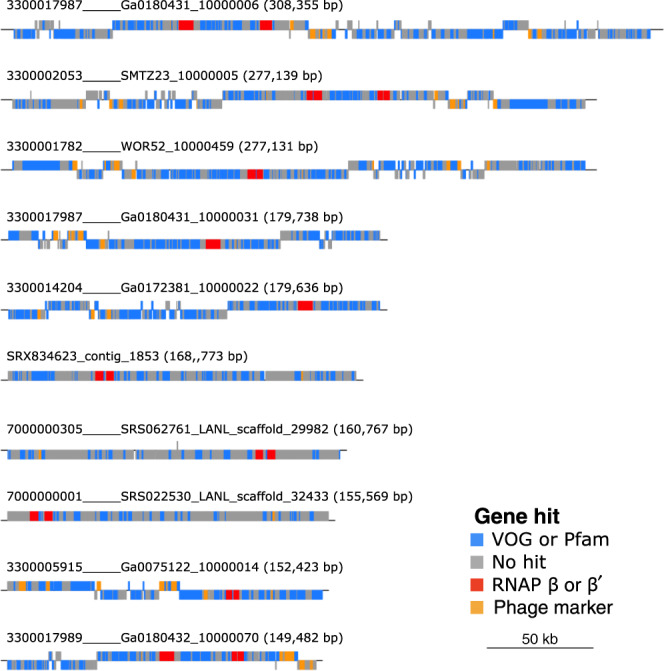
Genome plots of the ORFs of the ten longest mReC contigs. Above each plot is the contig name with its length in parentheses. Color corresponds to gene or database. Phage marker genes include baseplate wedge, portal proteins, capsids, terminases, and tail proteins. Scale bar corresponds to genome length of 50 kb.

We analyzed 1545 previously assembled metagenomic datasets^16^ and viral contigs available in the online viral sequence repository IMG/VR^17^ (see “Methods”). We compared all encoded proteins in these genomes and contigs against Hidden Markov Models constructed from cellular homologs of the β and β′ RNAP subunits (COG0085 and COG0086^18^) so that we could identify enzymes that have not diverged so far from their cellular homologs as to prevent robust sequence alignment and phylogenetic analysis (see “Methods” for details). In total, we identified 266 viral metagenomic contigs that encode both the β- and β′-subunits of RNAP. In diagnostic phylogenies, 97 contigs encoded RNAP subunits that clustered separately from homologs in cells and eukaryotic viruses (NCLDV) in a distinct deep-branching clade (Supplementary Fig. 1). These contigs also encoded viral signatures such as capsid, terminase, baseplate, wedge, portal, and tail proteins, indicating that they derive from *Caudovirales* (Fig. 1 and Supplementary Dataset 1). Moreover, three of these contigs were >200 kbp in length, suggesting they belong to “jumbo bacteriophage.” These contigs were identified in metagenomes that were sequenced from a variety of different aquatic, host-associated, and engineered environments, further suggesting they are widespread in the biosphere (Supplementary Fig. 2). These results are consistent with a recent large-scale metagenomic survey of viruses, which identified homologs of RNAP in several environmental bacteriophage sequences^15^. Given the unusual presence of multi-subunit RNAP in these *Caudovirales* contigs, which we refer to as mReC, we focused on them for purposes of this study.

### The mReC branch deeply within an RNAP Tree of Life

**Fig. 2:**
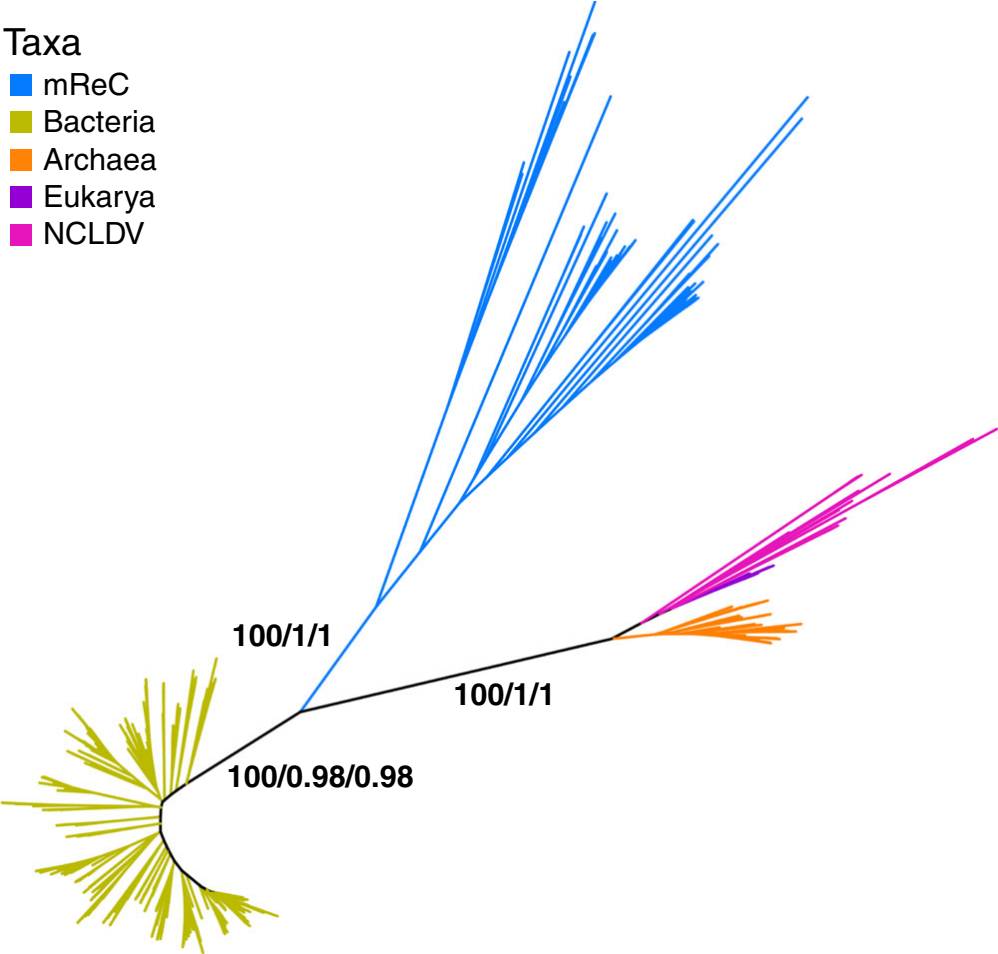
Unrooted phylogeny of concatenated RNAP β and RNAP β′ amino acid sequences. Homologs of β and β′ used for Eukarya were RPB2 and RPB1, and homologs B and A in Archaea, respectively. Phylogeny was constructed from the concatenated alignment of RNAP β and β′ of 589 taxa constructed using maximum likelihood with the amino acid substitution model LG + C60 + F + Γ4. Branch color corresponds to taxonomic group. Branch support values are from the left to right: ultrafast bootstrap from 1000 replicates reported as a proportion out of 100, relative Internode Certainty (IC) out of 1, absolute IC out of 1. Instructions for accessing full trees with support values can be found in the “Data Aavailability” section.

We constructed an unrooted phylogenetic tree of the mReC RNAP sequences with representative Archaea, Bacteria, Eukarya, and NCLDV (Supplementary Dataset 2) using maximum-likelihood analyses in IQ-TREE^19^, with amino acid substitution model LG + C60 + F + Γ4, a site-heterogeneous approach, which is particularly effective for estimating ancient divergences^20^, corrects for long branch attraction between divergent lineages, and is commonly used in deep phylogenetic studies^21,22^. The resulting tree revealed a distinct clustering of bacteriophage sequences on a branch separate from all other lineages (Fig. 2), with 100 ultrafast bootstrap support and an Internode Certainty (IC) of one for the monophyly of the mReC clade. The clustering of Archaea, Eukaryota, and NCLDV together and on a distinct branch from Bacteria is also consistent with previous studies^11^.

**Fig. 3:**
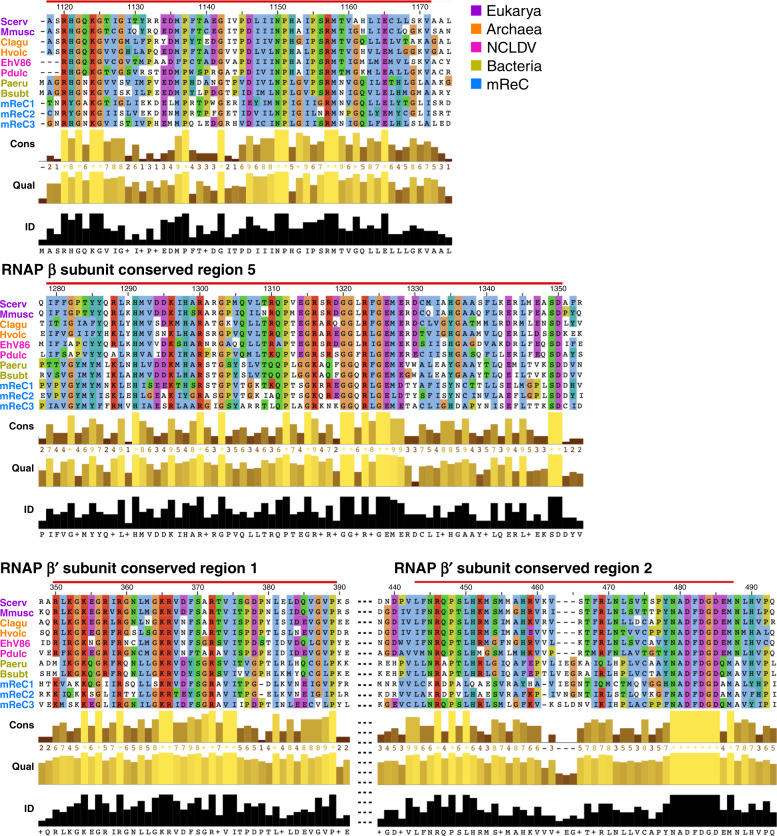
Highly conserved regions of the RNAP β- and β′-subunits alignment conserved across taxa. Eukarya homologs RPB2 and RPB1, Archaea homologs B and A, were used for β and β′, respectively. Scerv (*S. cerevisiae*), Mmusc (*Mus* musculus), Clagu (*Caldisphaera lagunensis*), Hvolc (*Haloferax volcanii*), Pdulc (*Pandoravirus dulcis*), Paeru (*Pseudomonas aeruginosa*), Bsubt (*Bacillus subtillis*), and mReC 1, 2, and 3 (multi-subunit RNAP-encoding *Caudovirales* 1, 2, and 3).

**Fig. 4:**
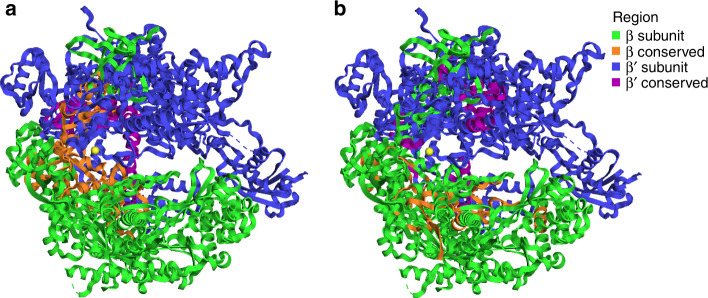
Conserved regions visualized in yeast RNAP protein structure. Image of RNAP β (RPB2 subunit in eukaryotes) and RNAP β′ (RPB1 subunit in eukaryotes) subunit structures of *S. cerevisiae* (PDBid: 2e2i)^24^. Colors correspond to subunit and conserved regions. **a** Regions conserved across all examined taxonomic groups (Supplementary Dataset 3) when amino acid sequences of β and β′ are aligned separately. **b** Regions conserved between β and β′ across all examined taxonomic groups when β and β′ are aligned to each other (Supplementary Dataset 3).

To ensure our unrooted phylogeny was based on a high-quality alignment of homologous RNAP regions, we manually inspected the β- and β′-subunit alignments, and identified eight highly conserved regions (Fig. 3 and Supplementary Fig. 3). These highly conserved regions were discerned based on both alignment conservation and quality (see “Methods”). Many of these regions corresponded to known conserved motifs in RNAP; within the β-subunit, these structures included the DNA-binding site, double-psi beta barrels, and the connector to the β′-subunit^23^. Within the β′-subunit, conserved regions included double-psi beta-barrel structures and the catalytic core^23^. This catalytic core hosts the active site, which coordinates a magnesium ion and contains the highly conserved NADFDG motif. Upon visualization in the structure of the yeast *Saccharomyces cerevisiae* RNAP II crystal structure (PDB ID: 2e2i; chain A of RPB1 and B of RPB2 corresponding to the β′- and β-subunits, respectively)^24^, we observed that highly conserved regions tended to be at the interface of the two subunits (Fig. 4a). This is consistent with selective pressure against mutations that interfere with the association and binding of the core subunits of the protein complex^12^.

In addition to using the LG + C60 + F + Γ4 site-heterogeneous model for phylogenetic construction, we also used several other approaches to correct for possible artefacts introduced by increased substitution rates in viral lineages, which could potentially result in long branch attraction. First, we constructed phylogenies from trimmed alignments using varying levels of stringency (positions with 10%, 30%, 50%, and 70% of gaps removed; Supplementary Fig. 4 and see “Methods”). We then evaluated the resulting alignment quality based on conservation, identity, and quality (Supplementary Dataset 3 and see “Methods”). Second, in addition to varying levels of trimming stringency, we removed up to 50% of fast-evolving sites in the RNAP alignment using the TIGER software^25^, which groups alignment sites based on their substitution rates. Removal of fast-evolving sites consistently maintained both the known monophyly of the clade grouping NCLDV, archaea and eukaryotic RNAP, and the distinct clustering of mReC RNAP from all cellular RNAP (Supplementary Fig. 5). Lastly, we also performed phylogenetic analysis using the PhyloBayes software^26^ to ensure that the results of maximum-likelihood and Bayesian approaches were consistent; using the alignment with 30% of gaps removed, we once again recovered a topology consistent with our other methods (Supplementary Fig. 6 and see “Methods” for details). Thus, by using a combination of different alignment quality checks and phylogenetic reconstruction methods, we provide evidence that the mReC RNAP sequences belong to a distinct lineage that likely have a common origin.

### The mReC form a distinct clade within the *Caudovirales*

**Fig. 5:**
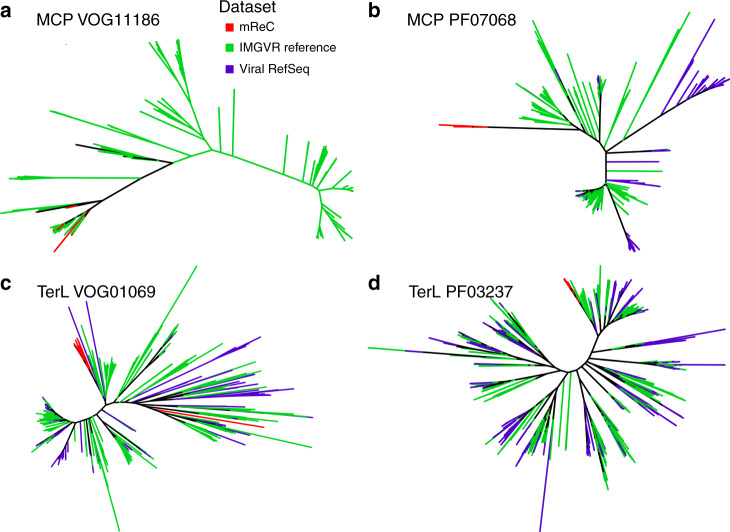
Unrooted phylogenies of mReC capsid and terminase proteins together with references. **a**, **b** Phylogenies of the protein sequences for the major capsid protein (MCP) and **c**, **d** terminase large subunit protein (TerL) from the VOG (**a**, **c**) and Pfam (**b**, **d**) databases. Branch color corresponds to the source of the sequences. Instructions for accessing full trees with support values can be found in the “Data availability” section.

To further investigate the monophyly of the mReC contigs, we performed phylogenetic analysis on other phage marker genes to ascertain if they supported the monophyly of mReC. We detected eight major capsid proteins (MCPs) and 31 large terminase subunit proteins (TerL) in these mReC (Supplementary Dataset 1). All MCP proteins had best matches to the same VOG and Pfam family (VOG11186 and PF07068, respectively), suggesting they have common evolutionary origins. For the TerL proteins, 27 of the 31 that had matches to the VOG database were classified to the same family (VOG01069); only 17 of these proteins had hits to TerL proteins in the Pfam database, all of which matched to the same family (PF03237). We reconstructed phylogenies of the MCP and TerL proteins, which showed that those proteins found within mReC tended to cluster together compared to available references in IMG/VR and Viral RefSeq, further suggesting they have common evolutionary origins (Fig. 5). The mReC proteins clade together with some references in IMG/VR that do not encode RNAP, but this is expected given that the contigs in this database are fragmented and may belong to complete genomes that encode RNAP. There were some exceptions to the trend of placement of mReC MCP and TerL proteins in the same region of these trees, such as a divergent TerL that is evident in our tree of VOG01069 references (Fig. 5c). Given that the mReC likely comprise a diverse clade of *Caudovirales*, it is likely that horizontal gene transfer has occurred within this group and other *Caudovirales* lineages at some point, which may explain this exception. Together with the observed distinct clustering of mReC RNAP, these results suggest that at least the majority of the mReC derive from a distinct clade of *Caudovirales* with a common evolutionary origin.

### Rooting analysis for the RNAP Tree of Life

Although the unrooted RNAP phylogenetic tree suggests that mReC RNAP originate from an ancient divergence from cellular homologs, the precise nature of these origins remain ambiguous as long as the tree remains unrooted. Previous studies have rooted the TOL using paralogous protein families that are the product of an ancient duplication that predates the divergence of the primary domains. In these approaches, which have variously used elongation factors, ATPase subunits, and aminoacyl-tRNA synthetases^27–29^, one gene family effectively serves as the outgroup for its paralog. As the β- and β′-subunits of RNAP are paralogous and originate from an ancient duplication^30^, we sought to use this approach to estimate a rooted tree.

**Fig. 6:**
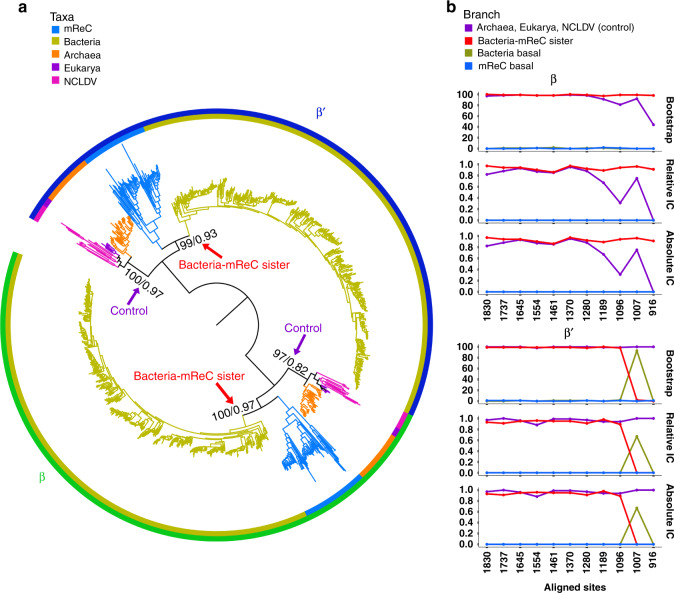
Paralogy-based rooting analysis of the RNAP tree. **a** Rooted tree of β- and β′-subunits. Homologs of the β- and β′-subunits used for Eukarya were RPB2 and RPB1, and homologs B and A used for Archaea, respectively. Amino acid sequences for each subunit were aligned to each other, and phylogeny was constructed using maximum likelihood with the amino acid substitution model LG + C60 + F + Γ4. Branch color and inner ring color strip corresponds to taxonomic group. Outer ring color strip corresponds to subunit β (green) and β′ (blue). At selected branches, first number refers to ultrafast bootstrap support of 1000 replicates reported as a percent out of 100 and the second number refers to relative Internode Certainty (IC) value out of 1. Arrows point to branches used to assess support of trees as fast-evolving sites were removed (see “Methods”). **b** Line plots of branch support corresponding to different hypotheses in the β (upper) and β′ (lower) clusters as fast-evolving sites are removed in steps of 5% until 50% of the fastest evolving sites are removed from the alignment (see “Methods”). Purple corresponds to branch support of Archaea, Eukarya, and NCLDV RNAP together. Blue line corresponds to the branch supporting mReC RNAP is basal to all other lineages considered. Yellow line corresponds to the branch supporting bacterial RNAP is basal to all other lineages considered. Red line corresponds to the branch supporting that mReC and bacterial RNAP diverged together prior to other groups. Instructions for accessing full trees with support values can be found in the “Data availability” section.

First, we aligned the β- and β′-subunits with each other and identified conserved regions (Supplementary Fig. 7). This alignment had markedly lower quality than alignments generated using only β and β′ individually (Supplementary Dataset 3), which is expected given these subunits arose from an ancient duplication event that likely took place before the divergence of Bacteria and Archaea. Nevertheless, distinct conserved regions were identified. Similar to the conserved regions found within the individual subunits, the regions shared between the β- and β′-subunits appeared to be at the interface of the two subunits (Fig. 4b). We then reconstructed a phylogeny using the LG + C60 + F + Γ4 amino acid substitution model in IQ-TREE, which we refer to as the β/β′ paralog tree. This tree revealed a topology in which Bacteria and mReC RNAP are sister clades in both the β- and β′-subunit regions of the tree (Fig. 6a). We proceeded to remove quickly evolving sites to evaluate the stability of this topology; we found that the Bacteria-mReC RNAP sister relationship was relatively stable (Fig. 6b). The monophyly of the Archaea–Eukarya–NCLDV branch was used as a control to assess when so many positions had been removed such that phylogenetic estimation became unreliable; the bootstrap and IC of this node remained high in almost all alignments. Interestingly, upon removing up to 45% of the fastest evolving sites, the most well-supported topology shifted in the β′-subunit such that Bacteria appear to have emerged prior to all other lineages (Fig. 6b), although this pattern was not shared in the β-subunit. This analysis provides some evidence that mReC RNAP were acquired at or near the diversification Bacteria, but results should be interpreted cautiously given they are derived from an alignment of highly divergent β- and β′-subunits; future analysis using additional sequences or incorporating structural information would potentially provide further insight.

One explanation for our observed results is that mReC acquired RNAP from cellular lineages in the distant past, potentially even prior to the diversification of the major bacterial phyla (i.e., from a proto-bacterial lineage). This interpretation must be made cautiously, however; while our concatenated β and β′ RNAP tree indicates that mReC RNAP forms a distinct clade separate from cellular lineages, the rooted β/β′ paralog tree is based on the alignment of highly divergent sequences and does not provide a definitive root. One may postulate an alternative scenario in which the mReC RNAP have an accelerated evolutionary rate that obfuscates phylogenetic analyses and potentially renders the resulting trees unreliable; indeed, other viruses encode YonO proteins or other homologs to RNAP subunits that have diverged considerably and cannot be robustly aligned to cellular homologs^13,14^. RNAP homologs from mReC still retain highly conserved regions that are readily alignable to cellular homologs, however (Fig. 3), suggesting that accurate phylogenetic assessment may still be possible for these proteins. Regardless, further analyses will be needed to definitively trace the evolutionary origin of these divergent *Caudovirales*-encoded RNAP genes.

## Conclusion

Here we provide phylogenetic evidence for the ancient acquisition of RNAP in a clade of *Caudovirales*, which is an important step in understanding the ancient evolution of this enzyme as well as the dynamic gene exchange between viruses and microbial life in the distant past. Although we originally suspected that multiple acquisitions of *Caudovirales* RNAP from their hosts would be the most likely explanation for the presence of these genes in bacteriophage, similar to what has recently been shown for phage-encoded ribosomal proteins^31^, the deep-branching placement of *Caudovirales* RNAP in our phylogenies implicates a single ancient acquisition within a distinct *Caudovirales* clade (Fig. 2), which we refer to as mReC. Phylogenies of capsid and terminase proteins in the mReC further supports their common evolutionary history. Using the paralogy of the β- and β′-subunits of RNAP, we assess the possibility that the divergence of these mReC sequences from cellular life occurred near the time of the divergence of Bacteria and the branch leading to Archaea, Eukarya, and NCLDV. Deep-branching nodes in our rooting analysis remain highly uncertain due to the highly divergent nature of the paralogous β and β′ alignment, however, and the results of analyses that rely on alignments of β- and β′-subunits remain speculative. Further work will be necessary to provide more insight into the precise timing at which these *Caudovirales* RNAP were acquired from cellular lineages.

It is likely that other bacteriophage groups have independently acquired RNAP from cellular lineages. For example, the human gut-associated crAssphage also encode a single protein that bears sequence motifs consistent with the fusion of β- and β′-subunits^32^; we were unable to identify any recognizable sequence homology of crAssphage RNAP to the COG0085 and COG0086 Hidden Markov Models (HMMs) we used in this study, however, indicating that the crAssphage enzyme is highly divergent and acquired independently from the mReC. Moreover, other phage have been found to encode a single-subunit YonO protein with similarity to the β′-subunit of RNAP^14^, although once again the highly divergent nature of these proteins hinders detailed phylogenetic analysis. It is not surprising that many divergent enzymes with either sequence or structural homology to RNAP subunits are present in the viral world considering the antiquity of this enzyme; indeed, structural homology has even been noted between RNAP subunits and eukaryotic RNA-dependent RNAP (RdRp) and archaeal replicative DNA polymerase^33^. Evolutionary analysis of ancient enzyme complexes such as multi-subunit RNAP can therefore yield important insight into ancient events in the evolutionary history of both cellular lineages and viruses.

## Methods

### Dataset selection and RNAP detection

As the majority of viruses in nature remain uncultured, we searched for bacteriophage RNAP in metagenomic nucleotide sequences from 1545 curated metagenomes^16^ (contigs > 10 kb) and IMG V/R release 1 July 2018 version 4 (contigs > 10 kb; 418,506 contigs)^17^, in addition to all cultured viral genomes with bacterial hosts available in viral RefSeq release 96^34^. We downloaded the nucleotide sequences from these datasets and predicted protein sequences with Prodigal^35^ (version 2.6.3). Default parameters were used for the RefSeq genomes and the -p meta option was used for the metagenomic sequences. Amino acid (aa) sequences were searched using HMMER3^36^ (v. 3.2.1) against HMM profiles of RNAP β and RNAP β′ from the Clusters of Orthologous Groups (COG) protein family database (2014 update)^18^, corresponding to COG0085 and COG0086, respectively (*E*-value 1*e* − 5). Metagenomic contigs and genomes retained for downstream analysis encoded both high-quality COG0085 and COG0086 matches that met the following criteria: minimum score of 80, minimum length of 800 aa, the presence of the conserved “NADxDGD” motif in COG0086, the presence of a predicted stop codon, and the absence of any “X” characters.

### Phylogenetic reconstruction and phage classification

To construct an RNAP phylogeny, we selected a diverse array of references (Supplementary Dataset 2). For eukaryote representation, genes corresponding to the β- and β′-subunits of RNAP II (RPB2 and RPB1 in the eukaryote nomenclature, respectively) were included, as this enzyme’s function most closely matches that of Bacteria and Archaea^30^. For initial diagnostic phylogenetic trees, these amino acid sequences were then input to the ete3^37^ (version 3.1.1) workflow in which a concatenated alignment was performed with Clustal Omega^38^ (v. 1.2.3), and a tree was inferred with FastTree^39^ (v. 2.1) using the standard_fasttree workflow and sptree_fasttree_all supermatrix. The tree was then visualized on the webserver iTOL^40^ (version 4.0; Supplementary Fig. 1). No RNAP sequences in bacteriophage genomes in viral RefSeq encoded RNAP subunits that met our criteria. Sequences encoding RNAP that did not cluster with RNAP of cells or eukaryotic viruses were considered belonging to putative bacteriophage. These sequences were then confirmed as viral based on presence of viral marker genes and enrichment of viral genes relative to cellular genes using the tool ViralRecall (contig mode, minimum score 0, https://github.com/faylward/viralrecall) (Supplementary Dataset 1). In addition, 48 of the 97 contigs encoded at least one phage hallmark gene (MCP, terminase, baseplate wedge, tail, and portal proteins), which were detected by searching against HMM profiles of these proteins in EggNog 5.0^41^, Pfam release 32^42^, and VOG (vogdb.org, downloaded 14 April 2020) databases (*E*-value 1*e* − 3) (full annotations can be found in Supplementary Dataset 1). Plots of the open reading frames of the largest ten contigs and their hits to the VOG and Pfam databases (using genoplotR^43^ (version 0.8.9) in R (version 3.5.1)^44^ using Rstudio (version 1.1.456)^45^ and Inkscape (v 0.92) (Fig. 1) revealed that the RNAP genes were typically surrounded by *Caudovirales* hallmark proteins.

To remove redundancy and lower computational load, 65 mReC from across the diversity of their encoded RNAP were selected to serve as a subset for subsequent phylogenetic analyses and alignment visualizations. Phylogenies of the β and β′ concatenated alignment were reconstructed using maximum likelihood in IQ-TREE v. 1.6.11 with the LG + C60 + F + Γ4 amino acid substitution model because mixed substitution rate models have been shown to be useful for phylogenetic reconstruction of divergent sequences^20^ and have been used in other studies for constructing deep phylogenetic relationships^21,22^. To estimate branch support, each tree was reconstructed with a 1000-replicate, ultrafast bootstrap approximation and RaXML^46^, to calculate absolute and relative IC values. IC has recently been proposed as a useful alternative to bootstrap support^47^ and these values give a measure of the support for a given topology compared to other well-supported alternatives.

### Alignment quality control

To improve the alignment for phylogenetic reconstruction, we trimmed the concatenated alignment of the β- and β′-subunits for positions containing gaps in over 10%, 30%, 50%, and 70% of the sequences using trimAl^48^ (version 1.2rev59). Alignments of each threshold were visualized in JalView^49^ and searched for regions of known conserved functions. Quality was assessed based on overall identity and conservation, which is calculated in JalView based on both identity and physio-chemical properties^50^. Additional metrics considered were output by the Alignment Manipulation and Summary tool^51^ with the summary command (Supplementary Dataset 3). Furthermore, we constructed concatenated β and β′ phylogenies with all of these trimming stringencies to assess their effect on results; ultimately we found that all trimming stringencies reliably recovered the deep-branching mReC RNAP clade (Supplementary Fig. 4). We report the results of 30% gaps removed in main text Figs. 2–4 and 6, and other trimming results are provided in Supplementary Fig. 4.

### Identification of highly conserved regions

To ensure confidence in the alignment quality and that the sequences of all taxa compared were homologs, we searched for regions of sequences conserved among all taxa. Sequences of the subunits β and β′ were aligned separately, and the alignment of each subunit was trimmed with a 30% gap threshold. Highly conserved regions within each subunit were manually distinguished based on consecutive positions of increased conservation and identity as calculated in the annotations file of the alignment output by JalView^49^ (version 2.11.1.0), the software in which the alignments were visualized (Fig. 3 and Supplementary Fig. 3). The mReC representatives in Fig. 3 correspond to the following: mReC1: 3300017989.....Ga0180432.10000070.a,mReC2: 3300000786.....WSSedA2C2DRAFT.1000021, mReC3: 3300017989.....Ga0180432.10000164. The average identity of these regions ranged from 66.039% to 81.832% and conservation ranged from 7.000 to 7.679 (Supplementary Dataset 3). The location of these regions was linked to function, when possible, based on the structural annotations of Iyer et al.^12^ and Sauguet^23^. Residues within the conserved regions were visualized on the structure of *S. cerevisiae* RNAP II (PDB: 2e2i chain A, chain B corresponding to the β′ homolog of RPB1 and β homolog of RPB2, respectively)^24^ using the graphical software PyMOL (The PyMOL Molecular Graphics System, Version 2.0 Schrödinger, LLC) (Fig. 4a).

### Topology testing with removal of fast-evolving sites

As viruses can have fast evolutionary rates, we reconstructed phylogenies after removing fast-evolving positions in the concatenated alignment that might have obscured the initial topology via long branch attraction. To identify fast-evolving sites in our concatenated β and β′ alignment, we used TIGER^25^ (version 1.02), which categorizes positions in an alignment into bins based on substitution rates. We set TIGER to bin each position in the alignment into 1000 different rates of substitution. We then reconstructed phylogenies after removal of fast-evolving sites; we did this in increments of 5% of full alignment length up to the fastest evolving 50% positions. Consistent with our initial methods, these trees were reconstructed using the LG + C60 + F + Γ4 amino acid substitution model and 1,000-replicates for ultrafast bootstrap approximation with IQ-TREE 1.6.11. Support for different topologies were assessed based on both ultrafast bootstrap values^52^ and IC values that were calculated with RAxML^46^ (version 8.2.12). These branch support values were plotted against alignment length in R with the package ggplot2^53^ (version 3.1.1) and results are presented in Supplementary Fig. 5.

### Long branch attraction assessment

In addition to alignment quality control and removal of fast-evolving sites, we performed the following analyses to ensure our results were robust and to limit the possible effect of Long Branch Attraction (LBA), which can manifest when highly divergent sequences are included in phylogenetic reconstructions.

First, we examined the similarity of the mReC RNAP β′-subunit to that of other proteins known to have common motifs, which included eukaryotic RdRp^12^ and crAssphage RNAP genes^13^. We performed an HMM search (*E*-value cutoff 10^−3^) of the RdRp proteins used in the study by Iyer at al.^12^ and the crAssphage RNAP proteins in the study Yutin et al.^13^ against COG0085 and COG0086 HMM profiles, and found no detectable sequence homology. Similarity between the eukaryotic RdRp was further examined by aligning the RdRp sequences with the COG0086 sequences (same as those used in Fig. 2) with Clustal Omega. The alignment was trimmed for 30% gapped regions using trimAl and a phylogeny was constructed using the LG + C60 + F + Γ4 amino acid substitution model and 1000 ultrafast bootstrap replicates in IQ-TREE. Branch support was estimated with RAxML for absolute and relative IC values. The resulting tree shows a clear case of LBA, with long branches of the RdRp clade within the NCLDV (Supplementary Fig. 8a).

As another test of LBA, we constructed another β′ phylogeny in which we included 12 bacteriophage sequences from Viral RefSeq that hit to COG0086, but did not meet our initial sequence filtering criteria, as this protein was typically fragmented into different genes, which resulted in low bitscores (details on the sequences included here can be found in Supplementary Dataset 2). We concatenated these fragmented sequences and aligned them with the RdRp and COG0086 proteins of taxa specified in Fig. 2 using Clustal Omega. We then reconstructed a tree with maximum likelihood in IQ-TREE using the LG + C60 + F + Γ4 amino acid substitution model and 1000 ultrafast bootstrap replicates, which yielded a tree that grouped the Viral RefSeq proteins with the RdRp (Supplementary Fig. 8b). The unstable branching of both RdRp and Viral RefSeq proteins suggests that LBA is a major issue for these sequences and provides justification for their exclusion from subsequent analyses.

To further assess whether the mReC monophyly held when only the mReC RNAP and Bacteria were compared alone, we constructed a concatenated alignment of the mReC and Bacteria RNAP in COG0085 and COG0086 amino acid sequences with the ete3 standard workflow. The resulting alignment was trimmed for 30% gapped positions, and we then constructed the phylogeny with maximum likelihood using the LG + C60 + F + Γ4 amino acid substitution model and 1000 ultrafast bootstrap replicates in IQ-TREE. Bootstrap and IC support values were calculated with RaXML. The resulting tree (Supplementary Fig. 9) once again recovered distinct clades of mReC and bacterial RNAP. This further suggests that the distinct clustering of mReC sequences is not due to long branch repulsion away from both bacterial and archaeal/eukaryotic sequences.

To test whether our results were maintained when using a different phylogenetic reconstruction method, we inferred the tree of Fig. 2 with Bayesian approaches using PhyloBayes 4.1c^26^ in which we ran two independent chains with the mixture model CAT + GTR, and the heterogeneity of site evolutionary rates were modeled using a gamma distribution with four categories. Supermatrices were recoded with the Dayhoff 6 scheme. The chains ran until convergence (maxdiff < 0.3) which was assessed with bpcomp using 1000 burn-in trees and checking every 10 trees to calculate posterior consensus. The consensus tree (Supplementary Fig. 6) maintained the topology observed in the maximum-likelihood reconstruction.

### Rooting analysis

Toward resolving a root in our RNAP phylogeny, we performed a rooting analysis based on the paralogy of the β- and β′-subunits. Leveraging the ancient gene duplication history of the β- and β′-subunits, one subunit can serve as the outgroup of the other subunit. First, we aligned the sequences of each subunit individually with Clustal Omega. We then trimmed this alignment with 30% gap threshold with trimAl. Next, we aligned these alignments of each subunit to each other with the profile-profile alignment option in Clustal Omega. To ensure the β- and β′-subunits are indeed paralogous and contain enough similarity for phylogenetic use, we visualized the alignment with JalView and identified regions conserved between the subunits and all taxa based on identity and conservation (Supplementary Dataset 3 and Supplementary Fig. 7). One of these regions corresponded to the double-psi beta-barrel structures conserved among both subunits^12,23,53^. All conserved regions were visualized on the structure of *S. cerevisiae* RNAP II (PDB: 2e2i chain A, chain B)^24^ in PyMOL (Fig. 4b).

To confirm the observed topology, as performed with the unrooted analysis, we removed the fastest evolving sites belonging to up to 50% of the positions in the alignment in increments of 5% with TIGER and trimAl. We then reconstructed phylogenetic trees of alignments with sites belonging to the fastest evolving rates incrementally with IQ-TREE using the LG + C60 + F + Γ4 amino acid substitution models and 1000 replicates for ultra-fast bootstrap approximation. Branch support for different topologies was inferred based on ultra-fast bootstrap values and IC values calculated with RAxML (Fig. 6a). These branch support values were recorded and plotted against alignment length in R with the package ggplot2^53^ (Fig. 6b).

### Major capsid protein and terminase diversity assessment

To explore the diversity of MCPs and large subunits of the terminase protein (TerL) in the mReC relative to other bacteriophage, we performed HMM searches of protein sequences from all mReC contigs, all contigs in IMGVR 2.0, and viral RefSeq genomes that had bacterial hosts against HMM profiles of bacteriophage MCP and TerL in the VOG and Pfam databases (Supplementary Dataset 1). Eight of the mReC contigs encoded MCPs and 31 encoded TerL. All mReC MCP proteins had best hits to the same VOG and Pfam families (VOG11186 and PF07068, respectively). Of all 17 mReC proteins with hits to a known TerL in the Pfam database, all had best hits to the same family (PF03237). Of 31 total mReC proteins had hits to a TerL family in VOG, 27 had best hits to VOG01069, and the remaining 4 had hits to different VOG TerL families (Supplementary Dataset 1). Phylogenetic trees including both mReC proteins and reference proteins with best matches to the same protein family were constructed to evaluate whether mReC proteins tended to cluster within the same clade. Due to the large number of reference proteins in IMGVR that had matches to the same Pfam and VOG protein families as the mReC MCP and TerL proteins, we randomly selected 500 reference proteins from the total hits in each of the IMGVR and RefSeq databases using seqtk subseq command^54^. We then generated alignments using Clustal Omega and phylogenetic trees using IQ-TREE (best model selected using the ModelFinder tool^55^, 1000 ultrafast bootstraps used; Fig. 5).

### Reporting summary

Further information on research design is available in the Nature Research Reporting Summary linked to this article.

## Supplementary information

Supplementary Information

Reporting Summary

Description of Additional Supplementary Files

Supplementary Data 1

Supplementary Data 2

Supplementary Data 3

## Data Availability

Sequences used included RefSeq: NCBI Reference Sequence Database release 96 (https://www.ncbi.nlm.nih.gov/refseq/) with accession numbers specified in Supplementary Dataset 2, Integrated Microbial Genomes/Virus release January 2018 (version 4) (https://genome.jgi.doe.gov/portal/IMG_VR/IMG_VR.home.html). Contigs used in this study are listed in Supplementary Dataset 2. Protein family HMM profiles were downloaded from the following databases with their version or release number in parentheses: Clusters of Orthologous Groups (COG, 2003, 2014 update; https://www.ncbi.nlm.nih.gov/COG/), eggNOG (v5.0; http://eggnog5.embl.de/#/app/downloads), Pfam (release 32; ftp://ftp.ebi.ac.uk/pub/databases/Pfam/releases/Pfam32.0/Pfam-A.hmm.gz), and Virus Orthologous Groups (VOG, downloaded 14 April 2020; vogdb.org). Amino acid sequences, alignments, phylogenetic trees, and tree branch support values are available at https://github.com/scubalaina/Bacteriophage_RNAP.

## Code availability

All software used was publicly available and cited with options reported in the “Methods.”
