# Supplementary material for: A distinct lineage of Caudovirales that encodes a deeply branching multi-subunit RNA polymerase
Source: Nat Commun. 2020 Sep 9;11:4506. doi: 10.1038/s41467-020-18281-3 (PMC7481178; doi:10.1038/s41467-020-18281-3)
Supplement: Supplementary file 1 — Supplementary Information [file 41467_2020_18281_MOESM1_ESM.pdf]

Supplementary Information for: ***A Distinct Lineage of* Caudovirales *that Encodes a Deeply Branching Multi-Subunit RNA Polymerase* by Weinheimer and Aylward**

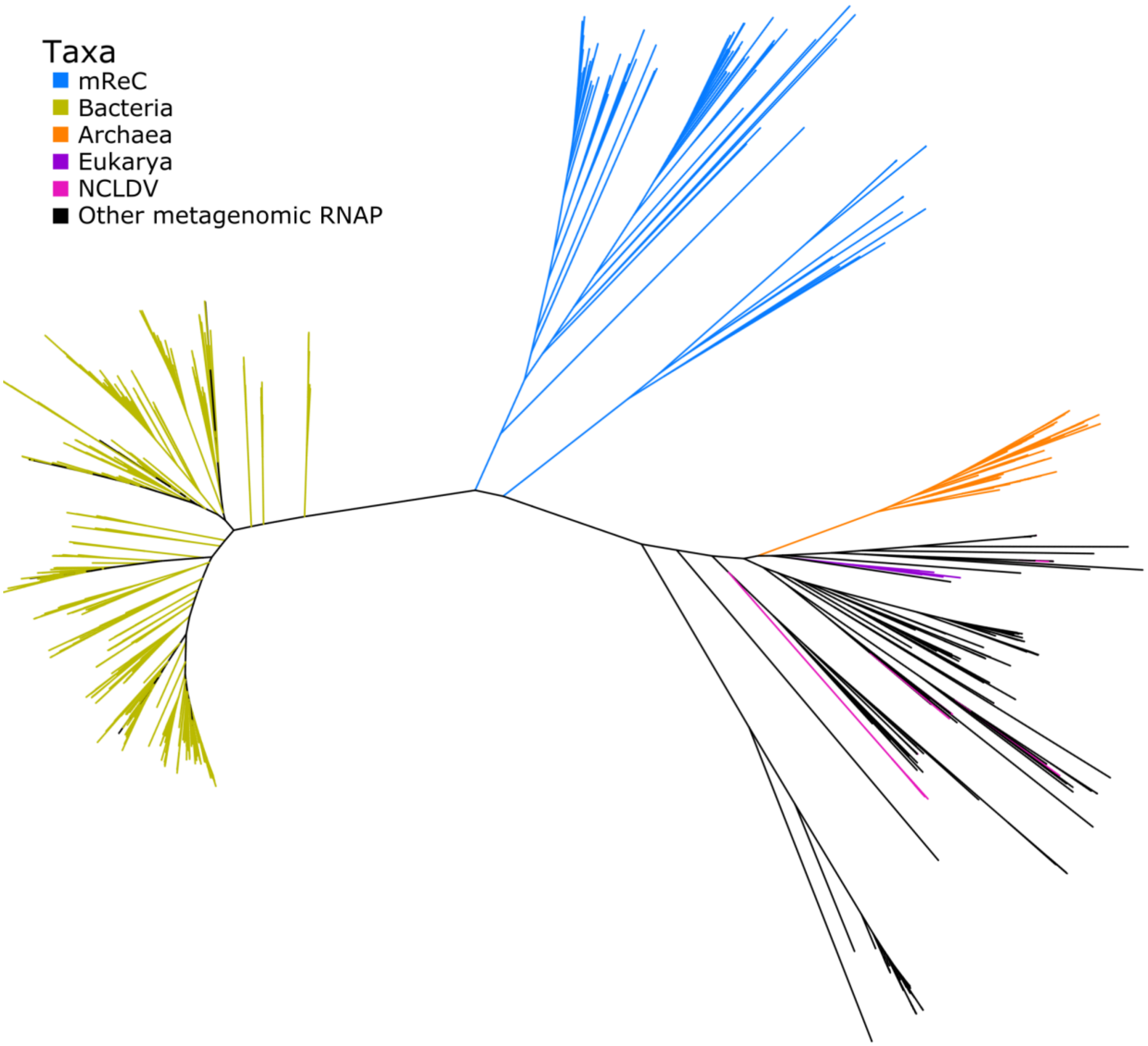

Supplementary Figure 1. Unrooted diagnostic tree constructed with FastTree of the concatenated β and β' amino acid sequence alignment (taxa in Dataset 1) for the initial identification of deep-branching bacteriophage RNAP. Branch color corresponds to taxa.

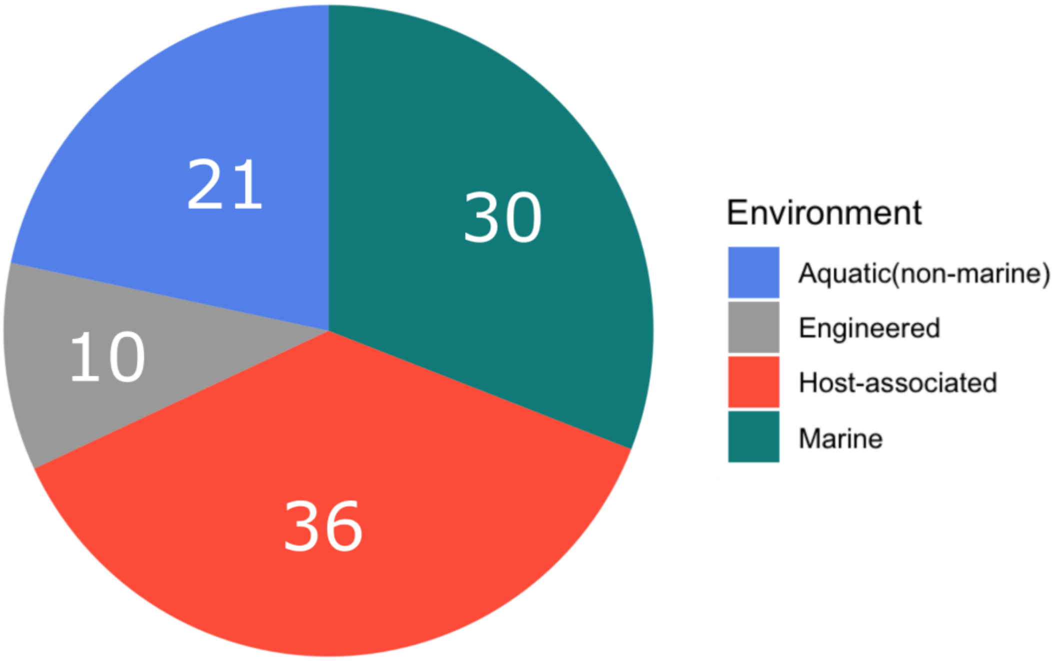

Supplementary Figure 2. Environmental distribution of the metagenomes from which mReC were identified (Dataset S3).

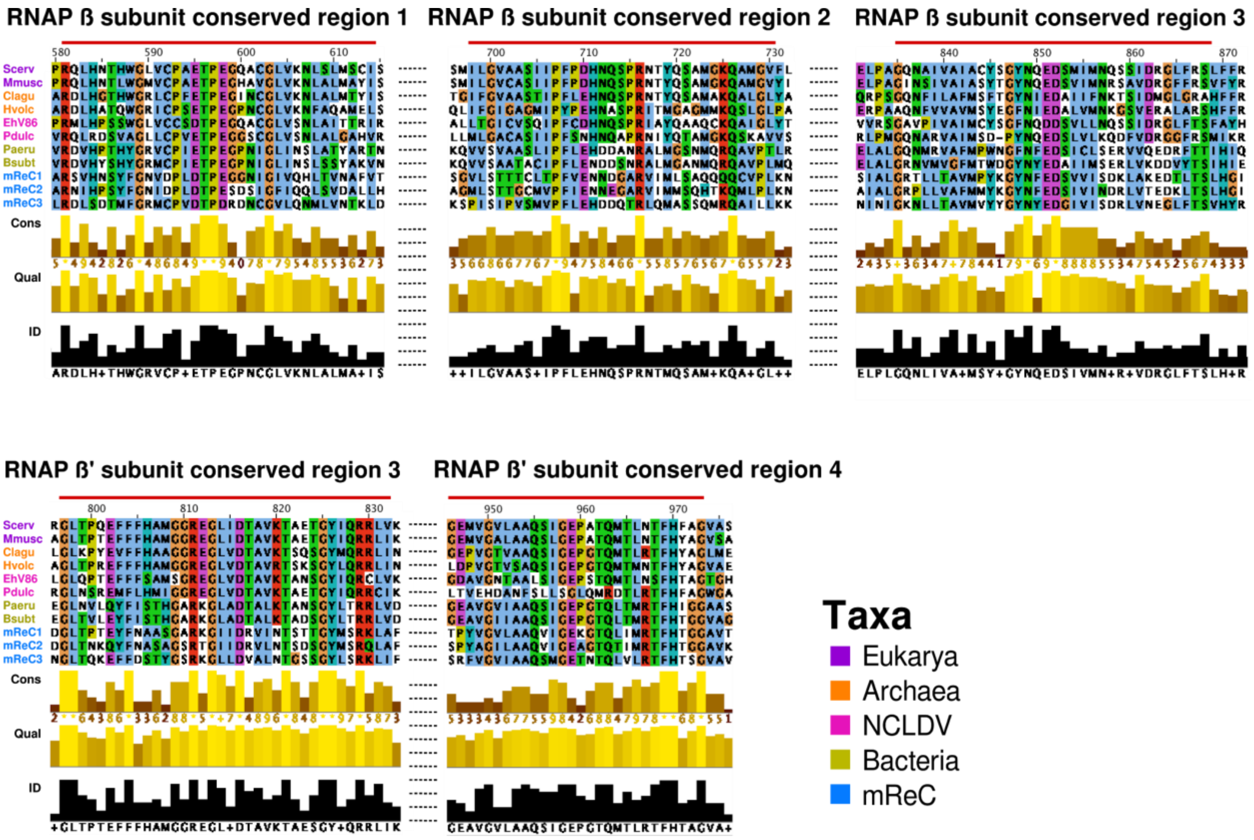

Supplementary Figure 3. Visualization of conserved regions, additional to those in the main text (Figure 2.) within each subunit of representative taxa as displayed in JalView with colors of residues corresponding to biochemical properties according to the Clustalx code. Colors of taxa on left correspond to taxonomic group according to Group table. Scerv (*S. cerevisiae*), Mmusc (*Mus* musculus), Clagu (*Caldisphaera lagunensis*), Hvolc (*Haloferax volcanii*), Pdulc (*Pandoravirus dulcis*), Paeru (*Pseudomonas aeruginosa*), Bsubt (*Bacillus subtillis*), mReC 1, 2, and 3 (multisubunit RNAP-encoding *Caudovirales* 1, 2, and 3).

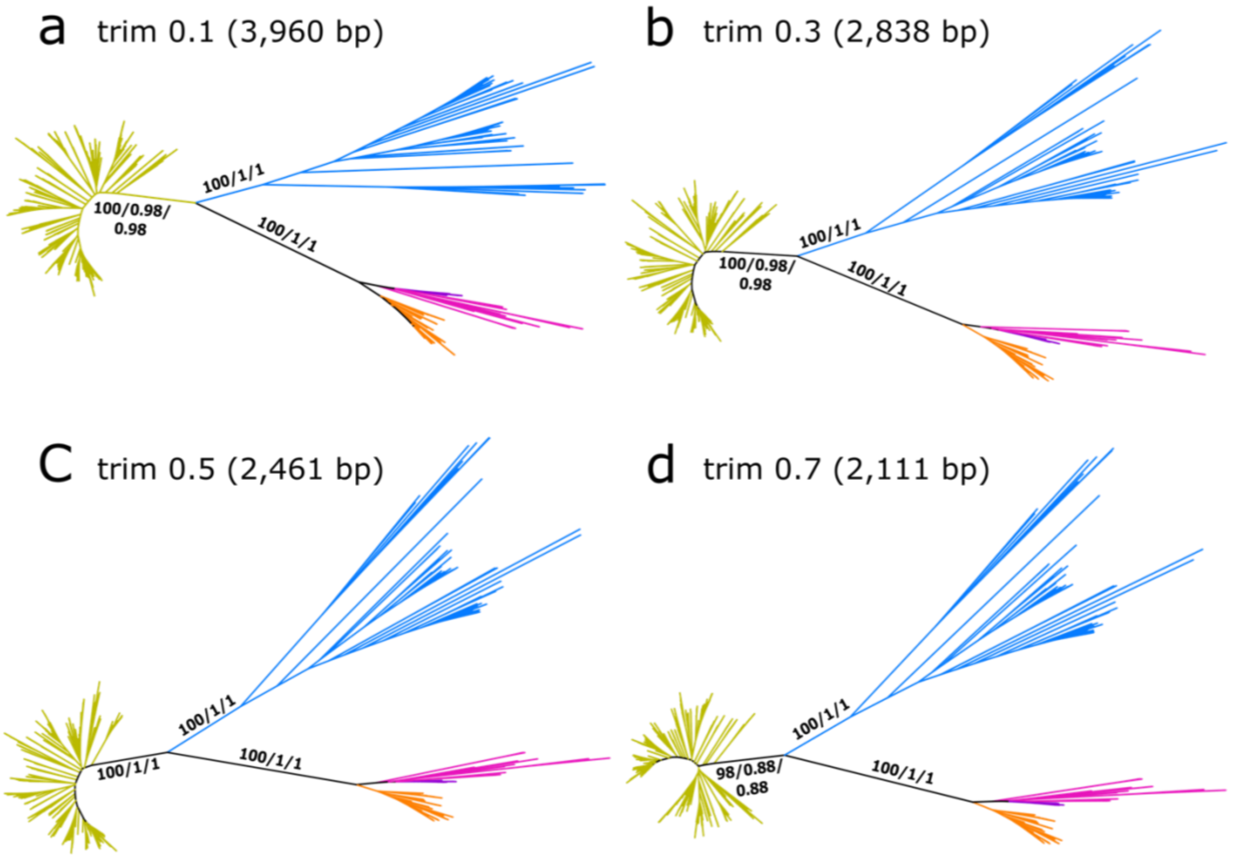

Supplementary Figure 4. Unrooted tree of concatenated β and β' amino acid sequence alignment constructed with maximum likelihood (LG+C60+F+Γ4 model in IQ-TREE). Same taxa as Figure 1. Branch colors correspond to taxonomy (yellow: Bacteria, blue: mReC, pink: NCLDV, purple: Eukarya, orange: Archaea). Each panel is a different gap-trimming stringency as a proportion out of 1 and alignment length is in parentheses. Branch support values from left to right: ultrafast bootstrap of 1000 replicates reported as a proportion out of 100, relative IC out of 1, and absolute IC values out of 1.

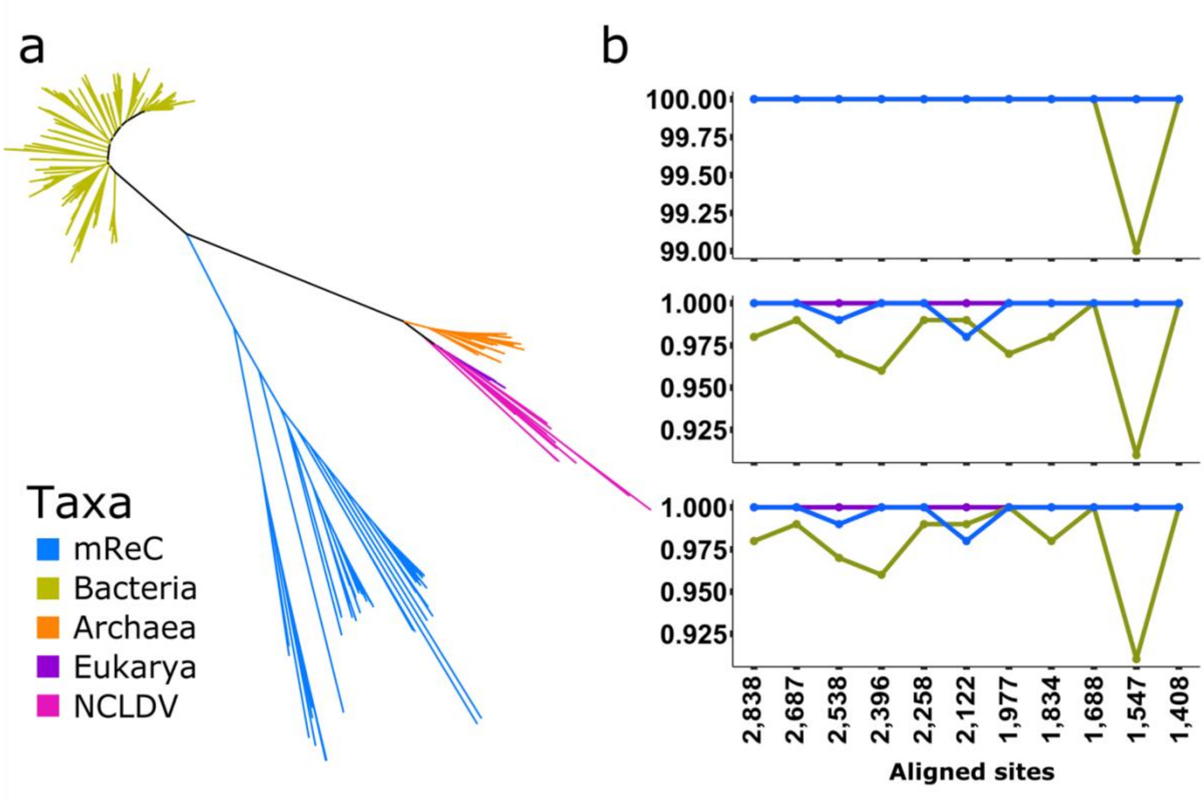

Supplementary Figure 5. a) Unrooted tree of concatenated β and β’ amino acid sequence alignment of taxa in Figure 1 constructed with maximum likelihood (LG+C60+F+Γ4 model in IQ-TREE). b) Results of removing fast-evolving sites; plots of branch support values, ultrafast bootstrap of 1000 replicates reported as a percent out of 100, relative IC out of 1, and absolute IC values out of 1, for the Archaeal, Eukaryotic, NCLDV clade (purple), Bacterial clade (yellow), and mReC (blue).

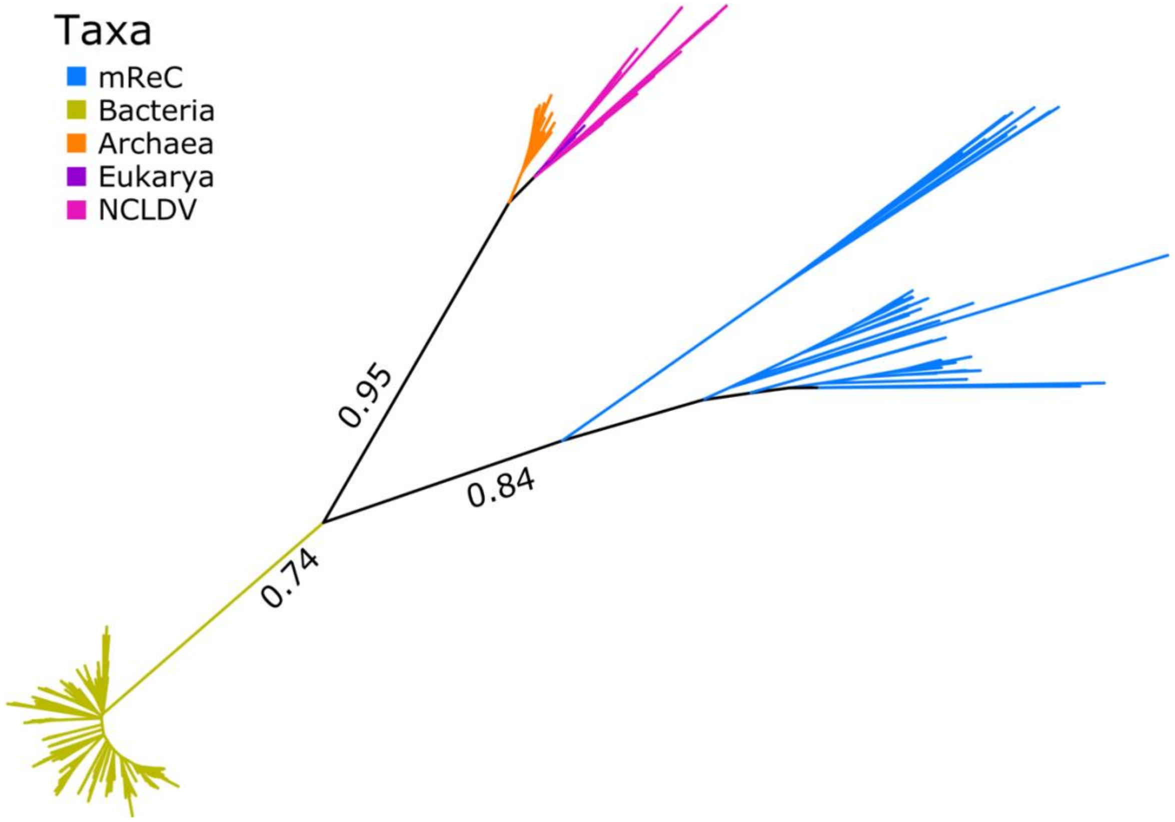

Supplementary Figure 6. Unrooted tree of concatenated β and β’ amino acid sequence alignment of taxa in Figure 1 reconstructed with the Bayesian approach implemented in PhyloBayes 4.1c (CAT-GTR-Γ4) consensus tree (maxdiff < 0.3). Branch support corresponds to posterior probabilities. Branch color corresponds to taxa.

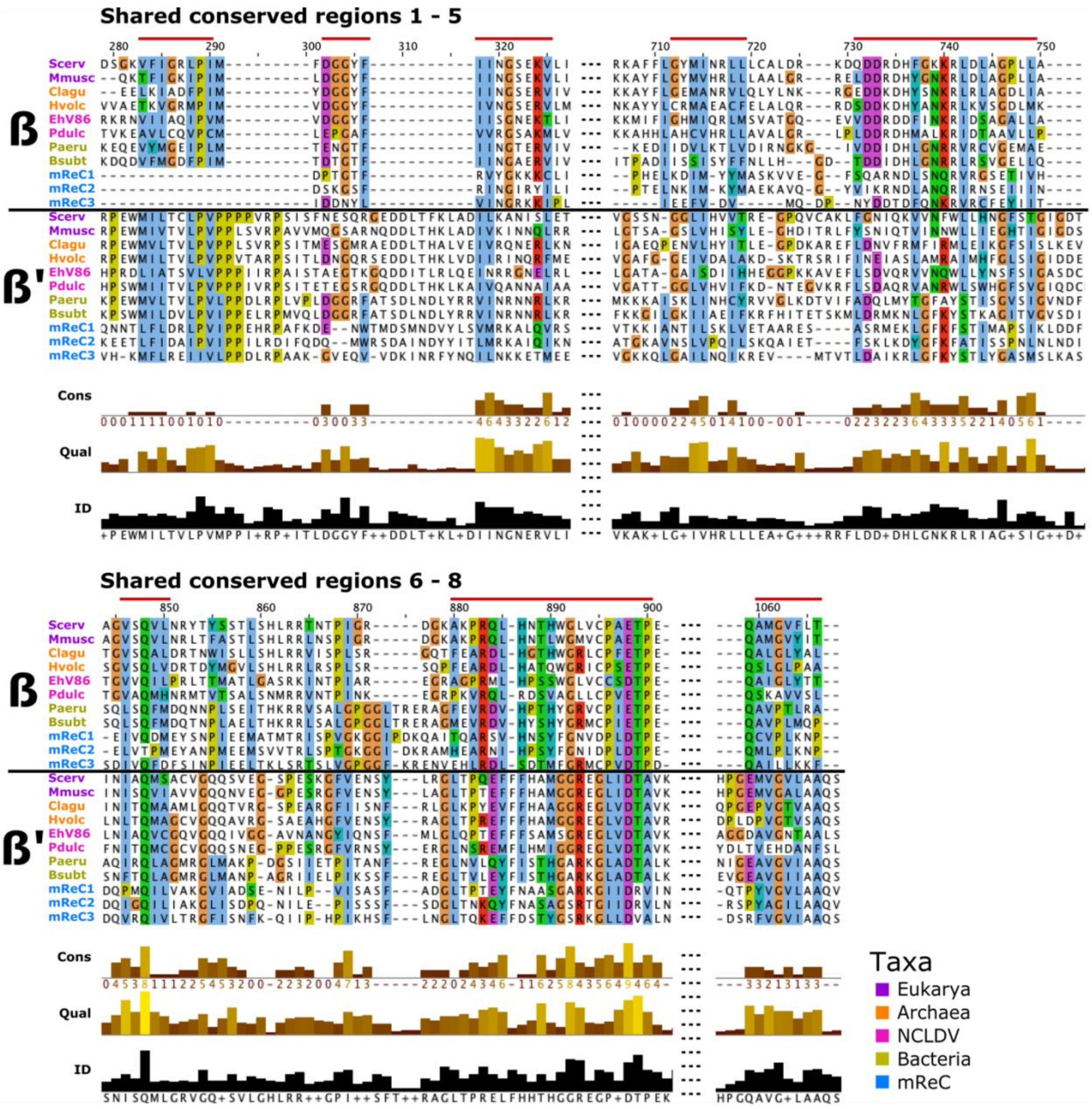

Supplementary Figure 7. Conserved regions between the aligned β and β' subunits of representative taxa as displayed in JalView Clustalx coloring (see Methods). For taxa abbreviations see Supplementary Fig. 2. Regions continue on next page.

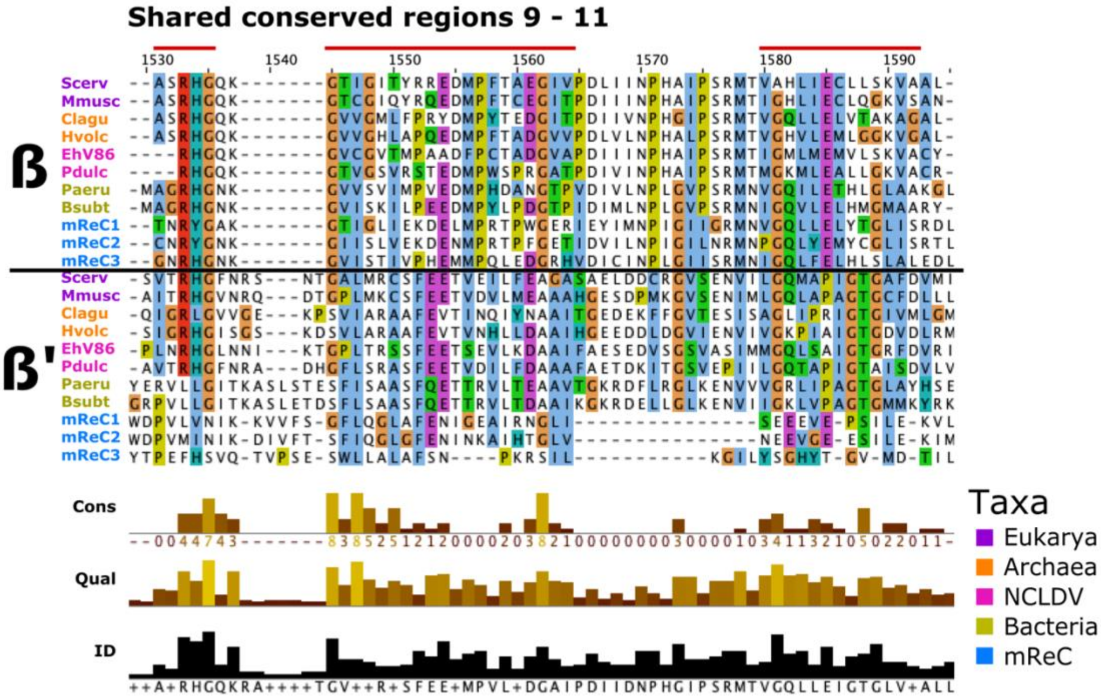

Supplementary Figure 7 continued.

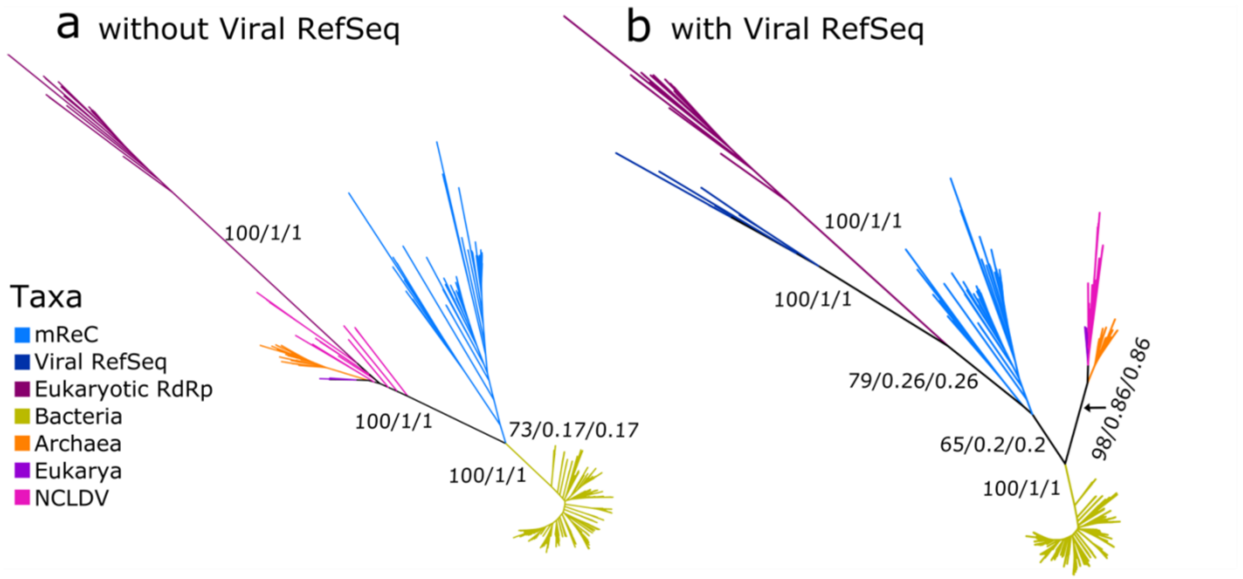

Supplementary Figure 8. a) Unrooted phylogeny of eukaryotic RNA-dependent RNA polymerase (RdRp) aligned with β' amino acid sequences of taxa in Figure 1 constructed with maximum likelihood (LG+C60+F+Γ4 model in IQ-TREE) with branch support values from left to right: ultrafast bootstrap support of 1000 replicates reported as a proportion out of 100, relative IC out of 1, and absolute IC out of 1. b) Phylogeny of sequences in a) aligned with Viral RefSeq β' amino acid sequences (Dataset 1).

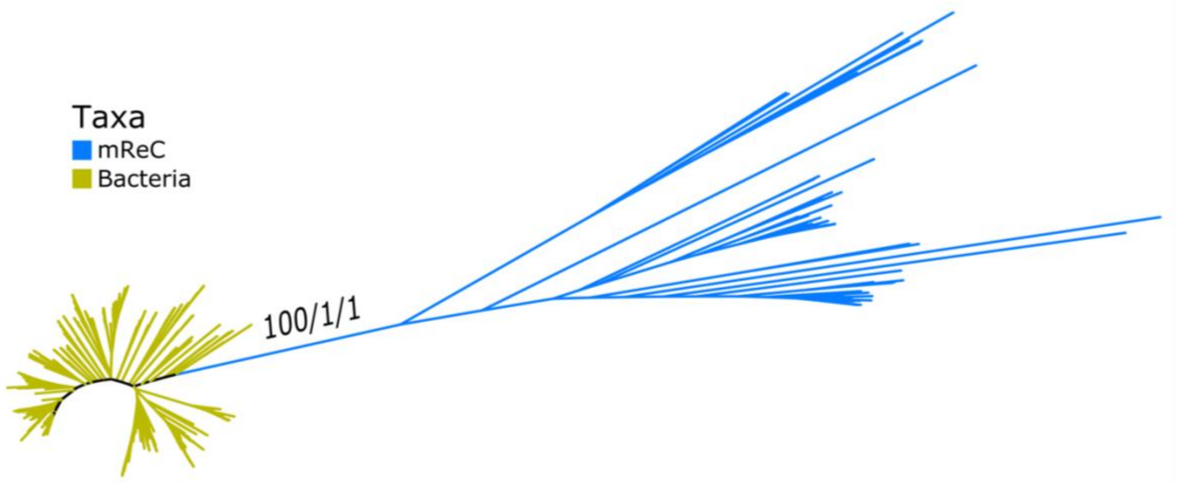

Supplementary Figure 9. Unrooted tree of concatenated alignment β and βamino acid sequences in Bacteria and mReC constructed with maximum likelihood (LG+C60+F+Γ4 model in IQTree) with branch support values from left to right: ultrafast bootstrap support of 1000 replicates reported as a proportion out of 100, relative IC out of 1, and absolute IC out of 1.
