# Supplementary material for: A distinct lineage of Caudovirales that encodes a deeply branching multi-subunit RNA polymerase
Source: Nat Commun. 2020 Sep 9;11:4506. doi: 10.1038/s41467-020-18281-3 (PMC7481178; doi:10.1038/s41467-020-18281-3)
Supplement: Supplementary file 2 — Reporting Summary [file 41467_2020_18281_MOESM2_ESM.pdf]

nature research

Corresponding author(s): Frank O. Aylward

Last updated by author(s): 08/07/2020

Reporting Summary

Nature Research wishes to improve the reproducibility of the work that we publish. This form provides structure for consistency and transparency in reporting. For further information on Nature Research policies, see Authors & Referees and the Editorial Policy Checklist .

Statistics

For all statistical analyses, confirm that the following items are present in the figure legend, table legend, main text, or Methods section.

n/a	Confirmed	
☒	☐	The exact sample size (*n*) for each experimental group/condition, given as a discrete number and unit of measurement
☒	☐	A statement on whether measurements were taken from distinct samples or whether the same sample was measured repeatedly
☒	☐	The statistical test(s) used AND whether they are one- or two-sided<br>*Only common tests should be described solely by name; describe more complex techniques in the Methods section.*
☒	☐	A description of all covariates tested
☒	☐	A description of any assumptions or corrections, such as tests of normality and adjustment for multiple comparisons
☒	☐	A full description of the statistical parameters including central tendency (e.g. means) or other basic estimates (e.g. regression coefficient) AND variation (e.g. standard deviation) or associated estimates of uncertainty (e.g. confidence intervals)
☒	☐	For null hypothesis testing, the test statistic (e.g. *F*, *t*, *r*) with confidence intervals, effect sizes, degrees of freedom and *P* value noted<br>*Give P values as exact values whenever suitable.*
☒	☐	For Bayesian analysis, information on the choice of priors and Markov chain Monte Carlo settings
☒	☐	For hierarchical and complex designs, identification of the appropriate level for tests and full reporting of outcomes
☒	☐	Estimates of effect sizes (e.g. Cohen's *d*, Pearson's *r*), indicating how they were calculated

Our web collection on statistics for biologists contains articles on many of the points above.

Software and code

Policy information about availability of computer code

Data collection	We downloaded sequences and databases used in this study by copying link addresses to the data directly from the host websites.
Data analysis	The following software and tools were used for analyses in alphabetical order with version in parentheses: Annotation Manipulation and Summary tool (AMAS; no version; https://github.com/marekborowiec/AMAS), Clustal Omega (1.2.3), ete3 (3.1.1), FastTree (2.1), genoplotR (0.8.9), ggplot2 (3.1.1), HMMER (3.2.1), Inkscape (0.92), IQ-TREE (1.6.11), iTOL (4.0), Jalview (2.11.1.0), PhyloBayes (4.1c), Prodigal (v2.6.3), PyMOL (2.1), R (3.5.1), RaXML (8.2.12), RStudio (1.1.456), seqtk (no version, https://github.com/lh3/seqtk), TIGER (1.02), trimAl (1.2rev59), and ViralRecall (https://github.com/faylward/viralrecall).

For manuscripts utilizing custom algorithms or software that are central to the research but not yet described in published literature, software must be made available to editors/reviewers. We strongly encourage code deposition in a community repository (e.g. GitHub). See the Nature Research guidelines for submitting code & software for further information.

Data

Policy information about availability of data

All manuscripts must include a data availability statement. This statement should provide the following information, where applicable:

- Accession codes, unique identifiers, or web links for publicly available datasets
- A list of figures that have associated raw data
- A description of any restrictions on data availability

Accession codes and sequence information used in this study are reported in Dataset S1 of the Supplemental. Additional data files are available on the GitHub repository: https://github.com/scubalaina/Bacteriophage_TOL .

Field-specific reporting

Please select the one below that is the best fit for your research. If you are not sure, read the appropriate sections before making your selection.

☐ Life sciences ☐ Behavioural & social sciences ☒ Ecological, evolutionary & environmental sciences

For a reference copy of the document with all sections, see nature.com/documents/nr-reporting-summary-flat.pdf

Ecological, evolutionary & environmental sciences study design

All studies must disclose on these points even when the disclosure is negative.

Study description	The phylogenetic relationship between cellular multi-subunit DNA-directed RNA polymerase (RNAP) was assessed with bacteriophage-encoded RNAP, using sequences of the two largest subunits.
Research sample	Data in this study were downloaded from NCBI RefSeq, IMG/VR, and Parks et al. 2017 study. HMM protein family databases downloaded included COG, Pfam, VOG, and eggNOG. See the Data availability section for details on version and links.
Sampling strategy	We examined the sequence databases RefSeq, IMG/VR, and Parks et al. 2017 for either bacteriophage RNAP or reference RNAP. These datasets were also searched for MCP and TerL proteins homologous to those encoded by the mReC contigs. We believe these datasets provided a thorough representation of RNAP, MCP, and TerL diversity in nature.
Data collection	Both Aylward and Weinheimer downloaded the sequence data and tools from the appropriate online repository using wget.
Timing and spatial scale	This study examines diversity of extant genetic sequences and does not involve time or spatial variables.
Data exclusions	No data was excluded from analyses.
Reproducibility	Procedure for bioinformatic replication is available in the Methods.
Randomization	We examined datasets in full for mReC RNAP, and thus, randomization was not required. Likewise, for phylogenetic analyses, we selected representative sequences that appropriately spanned the diversity of RNAP, and thus, randomization was not appropriate.
Blinding	This study did not include living subjects that would need blinding.

Did the study involve field work? ☐ Yes ☒ No

Reporting for specific materials, systems and methods

We require information from authors about some types of materials, experimental systems and methods used in many studies. Here, indicate whether each material, system or method listed is relevant to your study. If you are not sure if a list item applies to your research, read the appropriate section before selecting a response.

Materials & experimental systems

n/a	Involved in the study	
☒	☐	Antibodies
☒	☐	Eukaryotic cell lines
☒	☐	Palaeontology
☒	☐	Animals and other organisms
☒	☐	Human research participants
☒	☐	Clinical data

Methods

n/a	Involved in the study	
☒	☐	ChIP-seq
☒	☐	Flow cytometry
☒	☐	MRI-based neuroimaging
