# Supplementary material for: A distinct lineage of Caudovirales that encodes a deeply branching multi-subunit RNA polymerase
Source: Nat Commun. 2020 Sep 9;11:4506. doi: 10.1038/s41467-020-18281-3 (PMC7481178; doi:10.1038/s41467-020-18281-3)
Supplement: Supplementary file 3 — Description of Additional Supplementary Files [file 41467_2020_18281_MOESM3_ESM.pdf]

Description of Additional Supplementary Files

File name: Supplementary Dataset 1

Description: Information on bacteriophage-specific analyses (i.e. MCP/TerL trees, ViralRecall output). First sheet contains a description for each sheet in file.

File name: Supplementary Dataset 2

Description: Taxonomic and protein information on sequences examined for RNAP phylogenetic analyses. First sheet contains a full description for each sheet in file.

File name: Supplementary Dataset 3

Description: Statistics on the quality of alignments and conserved regions. First sheet contains description for each sheet in file. Yeast refers to the amino acid sequences of *Saccharomyces cerevisiae.*
